# Modulation of lung inflammation by vessel dilator in a mouse model of allergic asthma

**DOI:** 10.1186/1465-9921-10-66

**Published:** 2009-07-17

**Authors:** Xiaoqin Wang, Weidong Xu, Xiaoyuan Kong, Dongqing Chen, Gary Hellermann, Terry A Ahlert, Joseph D Giaimo, Stephania A Cormier, Xu Li, Richard F Lockey, Subhra Mohapatra, Shyam S Mohapatra

**Affiliations:** 1 Clinical Laboratory Center of First Affiliated Hospital, Xi'an Jiaotong University College of Medicine, Xi'an, PR China; 2 Division of Allergy and Immunology, University of South Florida, Tampa, FL 33612, USA; 3 Division of Endocrinology, Department of Internal Medicine, University of South Florida, Tampa, FL 33612, USA; 4 Department of Pharmacology and Experimental Therapeutics, Louisiana State University Health Sciences Center, New Orleans, LA 70112, USA; 5 VA Hospital Medical Center, Tampa, FL 33612, USA

## Abstract

**Background:**

Atrial natriuretic peptide (ANP) and its receptor, NPRA, have been extensively studied in terms of cardiovascular effects. We have found that the ANP-NPRA signaling pathway is also involved in airway allergic inflammation and asthma. ANP, a C-terminal peptide (amino acid 99–126) of pro-atrial natriuretic factor (proANF) and a recombinant peptide, NP73-102 (amino acid 73–102 of proANF) have been reported to induce bronchoprotective effects in a mouse model of allergic asthma. In this report, we evaluated the effects of vessel dilator (VD), another N-terminal natriuretic peptide covering amino acids 31–67 of proANF, on acute lung inflammation in a mouse model of allergic asthma.

**Methods:**

A549 cells were transfected with pVD or the pVAX1 control plasmid and cells were collected 24 hrs after transfection to analyze the effect of VD on inactivation of the extracellular-signal regulated receptor kinase (ERK1/2) through western blot. Luciferase assay, western blot and RT-PCR were also performed to analyze the effect of VD on NPRA expression. For determination of VD's attenuation of lung inflammation, BALB/c mice were sensitized and challenged with ovalbumin and then treated intranasally with chitosan nanoparticles containing pVD. Parameters of airway inflammation, such as airway hyperreactivity, proinflammatory cytokine levels, eosinophil recruitment and lung histopathology were compared with control mice receiving nanoparticles containing pVAX1 control plasmid.

**Results:**

pVD nanoparticles inactivated ERK1/2 and downregulated NPRA expression in vitro, and intranasal treatment with pVD nanoparticles protected mice from airway inflammation.

**Conclusion:**

VD's modulation of airway inflammation may result from its inactivation of ERK1/2 and downregulation of NPRA expression. Chitosan nanoparticles containing pVD may be therapeutically effective in preventing allergic airway inflammation.

## Background

Asthma is a complex disease, characterized by reversible airway obstruction, airway hyperresponsiveness and chronic airway inflammation. According to the Third National Health Nutrition Examination Survey, about 54% of the US population is allergic to one or more allergens, and over the last two decades, the rates of asthma have increased worldwide [1]. Current pharmacologic treatments for asthma include bronchodilating beta2-agonists and antiinflammatory glucocorticosteroids. These agents act only on symptoms and do not target the main cause of the disease, the generation of pathogenic Th2 cells [2-5]. Hence, there is a continued search for novel agents against allergy and asthma.

The family of natriuretic hormone peptides has broad physiologic effects including vasodilation, cardiovascular homeostasis, sodium excretion and inhibition of aldosterone secretion. There have been several reports demonstrating involvement of the atrial natriuretic peptide (ANP) signaling pathway in immunity in the heart and lung [6]. The natriuretic peptide prohormone is a polypeptide of 126 amino acids that gives rise to four peptides: long acting natriuretic peptide (LANP, amino acids 1–30), vessel dilator (VD, amino acids 31–67), kaliuretic peptide (KP, amino acids 79–98) and atrial natriuretic peptide (ANP, amino acids 99–126) [7]. In contrast to ANP, the N-terminal proANP peptides (LANP, VD, KP) are slowly metabolized and their plasma concentration is higher than ANP consistent with their important role in electrolyte balance and regulation of vascular tone. ANP and its principal receptor, NPRA, have been extensively studied in terms of cardiovascular effects [8]. ANP signals primarily through NPRA by increasing cGMP and activating cGMP-dependent protein kinase (PKG). Activated PKG turns on ion transporters and transcription factors, which together affect cell growth and proliferation, and inflammation [6]. NPRA is widely expressed in the lung and has been associated with allergic inflammation and asthma [9-11].

We have reported that both ANP and NP73-102 showed bronchoprotective effects [12,13]. Expression of NP73-102 induced constitutive nitric oxide production and decreased activation of a number of transcription factors including nuclear factor kappa B in human epithelial cells [13]. However, there is no report of the functions of the N-terminal proANP peptides including LANP, VD and KP in modulating lung inflammation. In this report we show that overexpression of VD attenuates airway inflammation in a mouse model of allergic asthma. The effects of VD on airway inflammation may result from its inactivation of ERK1/2 and downregulation of NPRA expression.

## Methods

### Mice

BALB/c mice were purchased from Harlan Sprague Dawley, Inc. and maintained under specific pathogen-free conditions within the vivarium at Louisiana State University Health Sciences Center (New Orleans, LA) or at the University of South Florida (Tampa, FL). Sentinel mice within each colony were monitored and were negative for specific known mouse pathogens. All animal protocols were prepared in accordance with the Guide for the Care and Use of Laboratory Animals (National Research Council, 1996) and approved by the Institutional Animal Care and Use Committee at Louisiana State University Health Sciences Center or at University of South Florida.

### Preparation of pVD chitosan nanoparticles

The cDNAs encoding VD were cloned between the *EcoR*I and *Xho*I sites of the mammalian expression vector pVAX1 (Invitrogene, CA) using standard molecular biology procedures. Similarly, we also constructed a plasmid, pMut, which expresses a mutated VD peptide with the reversed amino acid sequence of VD. Stocks of pVD, pMut and pVAX1 plasmids were prepared using Qiagene endotoxin-free Gigaprep kits (Qiagen, CA). We have developed a nanoparticle delivery system utilizing the polysaccharide chitosan that allows intranasal administration of peptides, plasmids, and drugs [14]. The nanoparticles protect the natriuretic peptide expression plasmids from nuclease degradation and improve delivery to cells. Complex coacervation of the DNA with chitosan (33 *k*D*a*, with 90% deacetylation, obtained from TaeHoon Bio (Korea) at a chitosan:DNA weight ratio of 1:3) was achieved by vortexing for 2 min at room temperature. Coacervates were used immediately after preparation or stored at 4°C.

### Analysis of ERK1/2 expression and NPRA in pVD-transfected cells by Western blot

A549 human alveolar carcinoma epithelial cells (ATCC, Manassas, VA) were grown in 6-well plates and transfected with 1 μg of pVD or pVAX1 using Fugene 6 under manufacture's instruction (Roche, NJ). To extract whole-cell protein, cells were harvested 48 hrs after transfection and resuspended in lysis buffer containing 50 mM HEPES, 150 mM NaCl, 1 mM EDTA, 1 mM EGTA, 10% glycerol, 0.5% NP-40, 0.1 mM phenylmethylsulfonyl fluoride, 2.5 μg/ml leupeptin, 0.5 mM NaF, and 0.1 mM sodium vanadate. Fifty μg of protein was subjected to sodium dodecyl sulfate-polyacrylamide gel electrophoresis on a 10% polyacrylamide gel and then transferred onto nitrocellulose membranes. Western blotting was performed using primary antibodies against extracellular signal-regulated kinase (ERK)1/2 according to the manufacturer's instructions (Cell Signaling Technology, Beverly, MA). For analysis of the effect of VD on NPRA expression *in vitro*, HEK-GCA (human embryonic kidney cells stably transfected with the natriuretic peptide receptor, GC-A, which is the same as NPRA) cells grown in 6-well plates were transfected with 1 μg of pVAX1, pVD or pMut. Differential expression of NPRA was detected by western blot using primary antibody against NPRA (Santa Cruz Biotechnology, CA).

### Luciferase assay to analyze the VD effect on NPRA promoter activity

Human embryonic kidney cells (HEK293; ATCC, Manassas, VA) were grown on 6-well plates and co-transfected with 1 μg of pVD plus 0.5 μg of pNPRA-Luc(-1575) which contains the 1575-bp fragment of NPRA promoter inserted upstream of the luciferase gene. In the control wells, cells were co-transfected with 1 μg of empty vector, pVAX1, and 0.5 μg pNPRA-Luc (-1575). Forty-eight hrs later, cells were washed with PBS, scraped off and suspended in 200 μl of luciferase assay lysis buffer (Promega, Madison, WI). Cell suspensions were kept on ice for 15 min and then vortexed for 15 sec before centrifugation for 1 min at 13,200 rpm. The supernatant was removed and aliquots were stored at -80°C. After protein concentration measurement, equal amounts of total protein from each transfection assayed for luciferase according to manufacturer's instructions (Pierce Biotechnology Inc., Rockford, IL).

### RT-PCR detection of NPRA expression in the lung

For analysis of the effect of VD on NPRA expression *in vivo*, three groups of mice (n = 4) were intranasally treated with 50 μl of chitosan nanoparticles containing 20 μg of pVAX1, pVD or pMut. Mice were sacrificed 48 hrs after nanoparticle treatment and lungs were collected. Approximately 100 mg of lung tissue from each mouse was treated with RNAlater (Invitrogen, CA), and total cellular RNA was extracted using Trizol reagent (Invitrogen, CA). RNA from each mouse was reverse transcribed and analyzed for NPRA by RT-PCR by using the following primers: NPRA-forward: 5'-cctgagtacttggaattcctgaagc-3'; NPRA-reverse, 5'-gttccacatccgctgagtgatgtt-3'. Mouse β-actin was used as housekeeping gene.

### Sensitization and induction of allergic airway response with OVA

**Figure 1:**
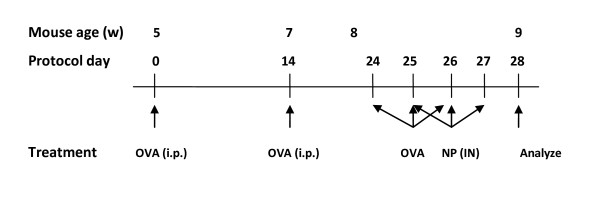
**Experimental schedule of sensitization and induction of allergic airway response**. Chicken ovalbumin was used to sensitize and challenge mice (n = 6–8 per group).

Mice (4–5 weeks old) were sensitized and challenged with chicken ovalbumin grade V (OVA; Sigma, St. Louis, MO) as previously described [15]. Briefly, mice were sensitized by an intraperitoneal injection (100 μl) of 20 μg OVA emulsified in 2 mg Imject Alum (Al [OH]3/Mg [OH]2; Pierce, Rockford, IL) on days 0 and 14 (Fig. 1). Mice were subsequently challenged with an OVA aerosol generated using an ultrasonic nebulizer (PariNeb Pro Nebulizer) from a 1% (wt/vol) OVA solution in saline for 20 min on days 24, 25, and 26. Nanoparticles (NPs) containing plasmids were administered intranasally (i.n.) on days 25, 26, and 27. Aerosolized OVA challenges were done six hours prior to i.n. NP treatment on days 25 and 26. The four groups of animals were: (1) naïve/pVAX1 (n = 8), exposed to vehicle and treated i.n. with 20 μg of NPs containing the pVAX1 control vector in 50 μl saline; (2) OVA/pVD20 (n = 6), sensitized and challenged with OVA and treated i.n. with 20 μg of NPs in 50 μl saline containing the pVD treatment vector; (3) OVA/pVAX1 (n = 8), sensitized and challenged with OVA and treated with empty vector; and (4) OVA only (n = 8), sensitized and challenged with OVA with no treatment. Pulmonary function testing was performed on day 28 (mice were 9 wks of age).

### Measurement of airway responsiveness to methacholine

Pulmonary resistance was measured using the forced oscillation technique as previously described [15]. Two sets of experiments that each included mice from all four experimental groups were performed on different days. Anesthetized animals were mechanically ventilated with a tidal volume of 10 ml/kg and a frequency of 2.5 Hz using a computer-controlled piston ventilator (Flexivent, SCIREQ; Montreal, Canada). Responses were determined in response to increasing concentrations of aerosolized methacholine (MeCh, at 0, 6.25, 12.5, and 25 mg/ml in isotonic saline). The single compartment model was used to determine airway resistance values and peak values obtained after each MeCh challenge were plotted [16].

### Modulation of lung inflammation by VD

To test the effects of VD on airway inflammation, a separate experiment was performed. BALB/c mice were divided into four groups (n = 8 per group). One group served as naïve control with no OVA sensitization and challenge while the second group received OVA sensitization and OVA challenge on days 18, 19, 20 and 21. Animals in the third group got OVA sensitization, OVA challenge and i.n. treatment with VD NPs on day 18, 19, 20 and 21. The last group was OVA sensitized and challenged, but treated with control NPs containing pVAX1. All mice were sacrificed on day 23 to collect bronchoalveolar lavage (BAL) fluid. Lungs were rinsed with intratracheal injections of PBS, perfused with 10% neutral buffered formalin, then removed, paraffin-embedded, sectioned at 20 μm and stained with hematoxylin and eosin (H & E). Lung homogenates for cytokine measurement were also prepared.

For differential cell enumeration, BAL fluid was centrifuged at 1,200 rpm for 5 min and the cell pellet was suspended in 200 μl of PBS and counted using a hemocytometer. The cell suspensions were centrifuged onto glass slides using a cytospin centrifuge at 1,000 rpm for 5 min at room temperature. Cytocentrifuged cells were air dried and stained with a modified Wright's stain (Leukostat, Fisher Scientific, Atlanta, GA) which allows differential counting of monocytes and lymphocytes. At least 300 cells per sample were counted by direct microscopic observation. For evaluation of proinflammatory cytokines, the levels of IL-2, IL-4, IL-5, IL-13, IFN-γ and TNFα in lung homogenates were measured using a mouse Th1/Th2 Cytokine CBA kit following the manufacturer's instruction (BD Bioscience, CA).

### Statistical analysis

All experiments were repeated at least once. The data are expressed as means ± SEM (standard error of the mean) and differences are considered significant at *p *< 0.05. Comparisons were done using the 2-tailed Student's *t *test or 2-way ANOVA with Bonferroni post-test.

## Results

### VD prevented ERK1/2 activation in A549 cells

**Figure 2:**
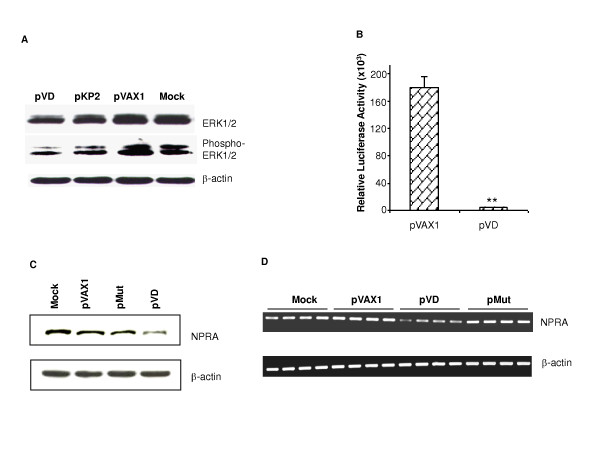
**pVD inactivates ERK1/2 and downregulates NPRA expression**. (A) A549 cells were transfected with pVD, pKP2 or pVAX1 control plasmids. Cells were collected 24 hrs after transfection. Expression of ERK1/2 and phospho-ERK1/2 was detected by western blot. (B) HEK293 cells grown on 96-well plates were cotransfected with 0.5 μg of pNPRA-Luc and 1 μg pVAX1 or pVD. Cells were lysed 48 hrs later and luciferase activity was measured in the lysates (p < 0.01). (C) Effect of VD on NPRA expression *in vitro*. HEK-GCA cells were transfected with pVAX1, pVD or pMut. NPRA expression was detected by western blot. Non-transfected cells were used as control. (D). Effect of VD on NPRA expression *in vivo*. NPRA mRNA expression was detected by RT-PCR in the lungs of mice intranasally treated with chitosan nanoparticles containing 20 μg of pVAX1 (n = 4), pVD (n = 4) or pMut (n = 4). Mice from the naïve group (n = 4) served as mock controls. All experiments were repeated, and the results of a representative experiment are shown.

Increased synthesis of nitric oxide (NO) during airway inflammation caused by induction of nitric oxide synthase-2 in several lung cell types may contribute to epithelial injury and permeability. Analysis of signaling pathways indicated ERK1/2 dephosphorylation as a possible contributing mechanism in NO-mediated HIF-1alpha activation [17]. We reported previously that that an N-terminal natriuretic peptide, NP73-102 (also termed KP2), which covers amino acids 73 to 102 of the ANP prohormone, had bronchoprotective and anti-inflammatory activity. Overexpression of NP73-102 increases NO and inactivates ERK1/2 in A549 cells [13]. In order to evaluate whether VD also inactivated ERK1/2, A549 cells were transfected with pVD. Transfection with pVAX1 alone was done as control. Expression of ERK1/2 and phosphorylation of ERK1/2 were analyzed by western blot. There was no significant change in expression of the total amount of ERK1/2 (Fig. 2A); however, significant dephosphorylation was observed in pVD-transfected A549 cells (Fig. 2A) which showed similarity between pVD- and pKP2- treated cells. Therefore, overexpression of VD inactivates ERK1/2.

### VD down-regulated NPRA expression

We have reported that NPRA plays a role in airway inflammation. Knockout of NPRA in mice resulted in less lung inflammation [11]. Inhibition of NPRA by small inferfering RNA against NPRA attenuated lung inflammation in a mouse model of asthma [10]. There is a feedback regulation of the circulating concentration of natriuretic peptides such that ANP decreases kaliuretic peptide and *vice versa *[18]. Although there is no direct evidence that VD interacts with NPRA, we investigated the effect of VD on NPRA expression. By luciferase assay, it was found that VD significantly decreased NPRA promoter activity up to 99% (p < 0.01, Fig. 1B). Downregulation of NPRA expression was also observed in HEK-GCA cells transfected with pVD (Fig. 2C) and by RT-PCR in the lungs of mice treated i.n. with pVD NPs (Fig. 2D) compared to pVXA1 and pMut controls. Further molecular mechanism studies are needed to demonstrate whether VD directly binds to the NPRA promoter or affects NPRA transcription though additional cellular factors.

### VD reduced airway hyperresponsiveness

**Figure 3:**
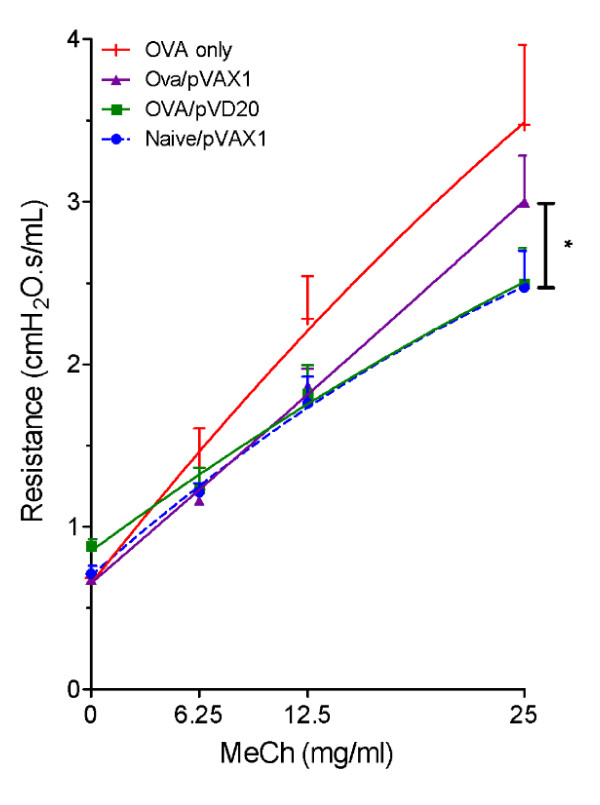
**VD prevents airway hyperresponsiveness in the mouse model**. Pulmonary resistance was measured using the forced oscillation technique. Mice from each group were treated with methacholine at increasing concentrations. Actual maximum resistance is displayed for each group. Mice given pVD chitosan nanoparticles had significantly lower resistance than those from the OVA control group or the group receiving pVAX1 control nanoparticles (p < 0.05).

Airway hyperresponsiveness (AHR) is one of the hallmarks of asthma, although it is regulated by a different set of genes from those controlling immunity and inflammation. To determine whether pVD can prevent AHR, pulmonary resistance was measured using the forced oscillation technique in response to increasing concentrations of aerosolized methacholine (MeCh). The baseline resistance values for each group were as follows: (1) naïve/pVAX1, 0.787 ± 0.242; (2) OVA/pVD20, 0.882 ± 0.093; (3) OVA/pVAX1, 0.676 ± 0.042; and (4) OVA, 0.686 ± 0.088. The baseline values were not statistically different from one another. Airway resistance of mice exposed to OVA alone was not different from that of mice exposed to OVA or the control pVAX1 plasmid. At 25 mg/ml MeCh, OVA-asthmatic mice treated with pVD had significantly decreased AHR compared to pVAX1-treated control mice exposed to OVA or control mice receiving OVA alone (Fig. 3). In a dose-response analysis, when mice were intranasally treated with NPs containing only 10 μg of pVD, less significant protection against AHR was observed (data not shown).

### VD treatment attenuated eosinophilia and lung pathology

**Figure 4:**
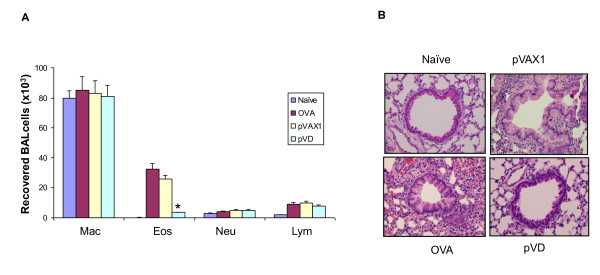
**VD attenuates lung inflammation in BALB/c mice**. (A) Mice were sensitized and challenged with OVA and then given nanoparticles containing pVD or control pVAX1 plasmids. Mice were sacrificed 48 hrs after the final treatment, and BAL fluids were collected for differential cell counts. Values are reported as mean ± SEM. Treatment with pVD significantly reduced eosinophil recruitment to the lungs compared to pVAX1 control (p < 0.05). Mac, macrophages; Eos, eosinophils; Neu, neutrophils; Lym, lymphocytes. (B) Lung sections from mice treated with VD nanoparticles also showed a substantial decrease in lung inflammation, goblet cell hyperplasia and infiltration of inflammatory cells compared to the non-OVA-challenged group or the group treated with pVAX1. All experiments were repeated and the results of a representative experiment are shown.

We also evaluated the effect of pVD NPs on lung inflammation in the mouse asthma model. After OVA sensitization and challenge with or without NP treatment, mice were sacrificed and BAL fluids were collected for eosinophil counts. Treatment with pVD NPs significantly reduced eosinophil recruitment to the lung (Fig. 4A) when compared to treatment with pVAX1 control NPs. To analyze the lung histopathology, the most direct indicator of airway inflammation, lungs were removed for H & E staining. Lung sections from mice treated with VD NPs showed a substantial decrease in inflammation, goblet cell metaplasia, and infiltration of inflammatory cells compared to the pVAX1 control group or the OVA control group with no treatment (Fig. 4B).

### VD treatment reduced TH2 inflammatory cytokines IL-4, IL-5 and IL-13

**Figure 5:**
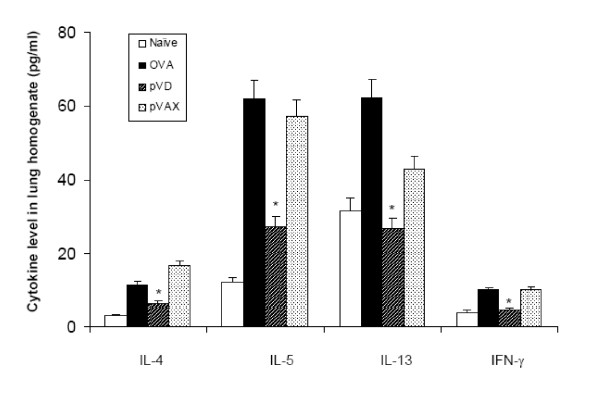
**VD reduces proinflammatory cytokines in lung homogenates**. Lungs from each group were collected and homogenized. Supernatants of the homogenates were used to measure proinflammatory cytokines with the mouse Th1/Th2 cytokine CBA kit. Significant reduction of IL-4, IL-5, IL-13 and IFN-γ were observed in the pVD nanoparticle-treated group compared to the OVA and pVAX1 nanoparticle-treated group (p < 0.05). All experiments were repeated at least once and the results of a representative experiment are shown.

Generation of pathogenic Th2 cells is the main cause of asthma. We measured a panel of proinflammatory cytokines in lung homogenates by using a mouse Th1/Th2 cytokine CBA kit. Significant reduction of IL-4, IL-5, IL-13 and INF-γ was observed in the pVD-treated group when compared to the pVAX1 control group (Fig. 5). However, there was no significant change in IL-2 and TNF-α after treatment with pVD NPs. Taken together, the observed changes in proinflammatory cytokines, AHR and lung pathology demonstrate that pVD NPs afford significant protection from airway allergic inflammation.

## Discussion

Here we demonstrate that intranasal treatment with pVD NPs decreases lung inflammation and protects against allergen-induced airway hyperresponsiveness. No VD-specific receptor has been identified, and the mechanism of how VD reduces airway inflammation is unknown. Here we show that VD inactivates ERK1/2 in A549 lung epithelial carcinoma cells, suggesting that VD may achieve its effect by interfering with the ERK1/2 signaling pathway [6,22,19-23].

We also tested whether VD attenuates lung inflammation through its interference with the ANP-NPRA signaling pathway. It has been reported that there is a feedback regulation of the circulating concentration of the N-terminal natriuretic peptide and C-terminal natriuretic peptide such that ANP decreases KP and *vice versa *[18]. We hypothesized that VD may behave like KP and that overexpression of VD may decrease the level of ANP and its receptor NPRA. We demonstrated that VD reduced NPRA promoter activity in a luciferase assay. Downregulation of NPRA expression was also confirmed both *in vitro *and *in vivo *by western blot and RT-PCR. Therefore, the observed attenuation of airway inflammation by VD is consistent with our previous report that NPRA-deficient mice or mice treated with siRNA for NPRA have less eosinophilia and lower levels of Th2-like cytokines compared to wild type mice [10,11].

Since no signal peptide sequence was placed in front of the VD ORF when pVD was constructed in our investigation, expressed VD remains inside the transfected cells (primarily lung epithelial cells). This differs from the normal biology of VD in which cleavage of the prohormone into N-terminal and C-terminal fragments occurs outside the cell. However, the intracellular expression of VD in lung cells may help us to meet our goal of developing a safe anti-inflammatory drug targeting the respiratory system. Because VD is a cardiovascular hormone, overexpression and circulation of VD may cause side effects. We will test the expression of a secreted form of VD in the future and it will be interesting to compare those results to the current data. Irrespective of the mechanism, the finding that ANP-NPRA is involved in the inflammatory immune response to allergens opens new avenues of research into the pathogenesis of allergic disease and asthma.

## Conclusion

The current study demonstrates that vessel dilator, VD, inactivates ERK1/2 and down-regulates NPRA expression. Inhibition of ANP-NPRA and ERK1/2 signaling pathways by VD affords bronchoprotection and anti-inflammatory activity; therefore, chitosan nanoparticles containing VD may be therapeutically effective in preventing allergic airway inflammation.

## Competing interests

The authors declare that they have no competing interests.

## Authors' contributions

SSM, SAC – design of experiments, interpretation of results. XL – analysis of the results. XW, WX, XK, DC, TAA, JDG – carrying out cell culture, western blot, luciferase assay. AHR and animal experiments. GH, RL, SM – writing and input in terms of discussion. All authors read and approved the final manuscript.
